# 纳米二氧化硅在人支气管上皮细胞内的亚细胞分布和遗传毒性

**DOI:** 10.3779/j.issn.1009-3419.2013.03.01

**Published:** 2013-03-20

**Authors:** 光强 赵, 云超 黄, 光剑 李, 森 李, 永春 周, 玉洁 雷, 小波 陈, 凯云 杨, 颖 陈, 堃 杨

**Affiliations:** 1 650118 昆明，昆明医科大学第三附属医院/云南省肿瘤医院胸心外科 Department of Cardiothoracic Surgery, the Third Affiliated Hospital of Kunming Medical University/The Tumor Hospital of Yunnan Province, Kunming 650118, China; 2 650118 昆明，昆明医科大学第三附属医院/云南省肿瘤医院肿瘤研究所 Research Institute of Tumor, the Third Affiliated Hospital of Kunming Medical University, Kunming 650118, China; 3 650032 昆明，昆明医科大学第一附属医院麻醉科 Department of Anesthesiology, the First Affiliated Hospital of Kunming Medical University, Kunming 650032, China

**Keywords:** 纳米二氧化硅, 支气管上皮细胞, 亚细胞分布, 遗传毒性, Silica nanoparticles, Human bronchial epithelial cells, Biological distribution, Genotoxicity

## Abstract

**背景与目的:**

纳米二氧化硅广泛应用于社会生产生活中，肺部是吸入暴露纳米二氧化硅的主要靶器官，因此，二氧化硅对肺部的生物毒性作用引起人们的广泛关注。本研究旨在探讨纳米二氧化硅在人支气管上皮细胞内的亚细胞分布和遗传毒性。

**方法:**

应用透射电子显微镜（transmission electron microscope, TEM）观察不同粒径二氧化硅在人支气管上皮细胞（immortalized human bronchial epithelium cells, BEAS-2B）内的亚细胞分布；应用单细胞凝胶电泳检测不同粒径二氧化硅处理BEAS-2B细胞24 h后的DNA损伤，了解不同粒径二氧化硅的遗传毒性作用。

**结果:**

透射电镜观察到微米二氧化硅不能进入细胞，纳米二氧化硅赋存在细胞质，纳米二氧化硅导致线粒体、内质网等细胞器损伤。纳米二氧化硅导致比微米二氧化硅更严重的DNA损伤（*P* < 0.05）。

**结论:**

二氧化硅的粒径决定二氧化硅颗粒物是否能进入细胞及在细胞内的分布，纳米二氧化硅对细胞遗传毒性比微米二氧化硅严重。

## Abstract (en)

**Background and objective:**

Silicon nanoparticles are widely used in daily life. Therefore, they attract increased attention because of their potential biotoxicity to the lungs when inhaled. The aims of this study are to explore the organism distribution and genotoxicity of silica nanoparticles in human bronchial epithelial cells (BEAS-2B).

**Methods:**

The biodistribution of silica with different particle sizes in human bronchial epithelial cells was observed by transmission electron microscopy (TEM). DNA damage was detected by single-cell gel electrophoresis (comet assay).

**Results:**

TEM revealed that SiO2 nanoparticles with different sizes can be uptaken by cells and be localized in the cytoplasm and the nucleus. Compared with micro-silica, nano-silica in BEAS-2B cells can inflict more severe DNA damage (*P* < 0.05).

**Conclusion:**

The particle size of silica nanoparticles can be used to determine their distribution in biological cells. Compared with micro-silica, nano-silica has higher genotoxicity.

随着纳米二氧化硅材料的出现和在生物医药工程、材料、化妆品等领域的广泛应用，肺部是吸入暴露纳米二氧化硅的主要靶器官，纳米二氧化硅对肺部的生物毒性作用引起人们的广泛关注。二氧化硅的致癌性国际上经历了数十年的争论。国际癌症研究组织（International Agency for Research on Cancer, IARC）于1987年宣布石英为动物致癌物、人类可疑致癌物，1996年10月又宣布将石英由动物致癌物升级为人类致癌物，2010年再次得到确认^[1]^。尽管IARC已将石英定为人类致癌物，但由于流行病资料结果的不一致以及其作用机制尚未阐明，国内外学者对此仍存有争议。

天然存在的游离二氧化硅粉尘的粒径大小不一。众多研究^[2-4]^表明，石英可以引起矽肺，但石英、矽肺和肺癌三者关系研究并不清楚，分析原因，可能是未考虑石英的粒径因素。纳米二氧化硅作为纳米颗粒，由于粒径小、比表面积大和不饱和键的存在，可能影响其在细胞内的亚细胞分布和自由基生成，造成比微米二氧化硅更大的遗传毒性。

本实验拟利用永生化人支气管生皮细胞（immortalized human bronchial epithelium cells, BEAS-2B）研究不同粒径纳米二氧化硅和微米二氧化硅的细胞内亚细胞分布和DNA损伤情况，来评价二氧化硅粒径对细胞遗传毒性的影响，为纳米二氧化硅的进一步遗传毒性研究提供一定理论依据。

## 1 材料与方法

### 1.1 材料

#### 1.1.1 纳米二氧化硅和微米二氧化硅

纳米二氧化硅：粒径60 nm±5 nm，30 nm±5 nm，15 nm±5 nm。纯度均>99.65%（上海晶纯实业有限公司）；微米二氧化硅：粒径大小1 µm，纯度>99.5%（上海晶纯实业有限公司）。

#### 1.1.2 细胞株及主要试剂

永生化人支气管上皮细胞株BEAS-2B（购自中国科学院典型培养物保藏中心昆明细胞库）。该细胞系主要用于筛选诱导或影响分化及致癌的化学或生物制剂。LHC-8培养基、DMEM高糖培养基（Invitrogen/USA）；胎牛血清（杭州四季青生物公司）；胰蛋白酶（Pittsburgh, PA, USA）；25%戊二醛（Merck/UK）；四氧化锇（EHSY/Hong Kong）；醋酸铀（北京恒业中远化工有限公司）；丙酮（深圳市华昌化工有限公司）；硝酸铅（太原市欣吉达化工有限公司）。

#### 1.1.3 实验室配置试剂

磷酸盐缓冲液（phosphate buffered-saline, PBS）(135 mM NaCl, 2.7 mM KCl, 1.5 mM KH_2_PO_4_, 8 mM K_2_HPO_4_, pH7.2）；0.1 M磷酸缓冲液的1%四氧化锇固定液（0.2 M磷酸缓冲液10 mL；2%四氧化锇10 mL）；柠檬酸铅液1.33 g；柠檬酸三钠1.76 g；去CO_2_重蒸水30 mL；剧烈振荡30 min，呈白色悬液时，加入8 mL的1% NaOH，悬液澄清后加重蒸水至50 mL，pH12.0，环氧树脂Epon618。

#### 1.1.4 主要仪器设备

超净工作台（中国海尔集团）；BBD6220型CO_2_培养箱、MEGFUGE 10.0型低温超速离心机（Heraeus/Germany）；8600型-80 ℃超低温冰箱、Biorad Model 450型酶标仪（Thermo/Germany）；-20 ℃低温冰箱（中国中科美菱集团）；1730MK型蒸气高压灭菌器（Tuttnauer/Israel）；CPA225D型电子分析天平（Sartorius/Germany）；U-LH100HG型倒置相差显微镜（OLYMPUS Optical/Japan）；DM4000B显微摄影系统、R型0.1 µm超薄切片机、DFC320数码相机及IM50摄影软件（Laica/Germany）；JEM-100CX Ⅱ型透射电子显微镜（日本电子公司）；XL30ESEM-TMP扫描电子显微镜（PHILPS/Holland）；Phoenix+DIM一体化能谱及电子背散射衍射仪（EDAX/USA）；TE-200U型荧光显微镜（NIKON/Japan）；流式细胞仪（Becton Dickinson FACScan/USA）；高通量彗星试验平台、彗星图象智能分析软件Comet A 1.0（bio-radCo/USA）；超声波分散仪（FS-60H, 130 W, 20 kHz, Fisher Scientific, Pittsburgh, PA, USA）。

### 1.2 方法

#### 1.2.1 BEAS-2B的培养

将BEAS-2B培养于LHC-8培养基中，培养条件为37 ℃、5%CO_2_的培养箱。每3天传代1次，传代后第3天换培养液。

#### 1.2.2 不同粒径二氧化硅DMEM培养基的配制

用分析天平分别称取微米二氧化硅（粒径1 µm）、纳米二氧化硅（粒径：60 nm、30 nm、15 nm），置于MK半自动型蒸气压力灭菌器中消毒30 min。在超净台里将消毒后的不同粒径二氧化硅分别用DMEM培养基配置成浓度为25 mg/mL、50 mg/mL和75 mg/mL，充分搅拌混匀，4 ℃储存备用。

#### 1.2.3 二氧化硅颗粒物悬浮溶液配制及处理BEAS-2B细胞

BEAS-2B细胞在含10%胎牛血清的Ham’s F-12（含L-谷氨酰胺Cellgro USA）培养基中培养，培养基中加入100 U/mL的青霉素和100 µg/mL的链霉素，在37 ℃、5%CO_2_培养基中孵化。取对数生长期的BEAS-2B细胞接种于96孔培养板中，5×10^4^个细胞/孔。每组设3个复孔，待细胞贴壁48 h后分别加入不同浓度、不同粒径的二氧化硅悬浮液共同培养。使细胞培养基中的刺激浓度分别达25 mg/mL、50 mg/mL和75 mg/mL。处理24 h后，检测细胞成活率变化情况。

#### 1.2.4 细胞成活率的测定

应用台盼蓝抗法检测细胞成活率，将细胞收集在用HBSS溶液配制的0.25%胎盼蓝溶液中，使最终溶液达1.5 mL并置于冰上，死亡或损伤的细胞被染成蓝色，有活性细胞不被染色，5 min后应用细胞计数器计算每个培养基中无活性细胞占总的细胞的百分比。从而得到活性细胞的比率。每组设3个复孔，每个试验设3个重复。

#### 1.2.5 透射电镜检测处理后BEAS-2B细胞内不同粒径二氧化硅的分布

透射电子显微镜观察不同粒径二氧化硅（浓度为50 mg/L）处理24 h后，二氧化硅颗粒物在BEAS-2B细胞内赋存分布和细胞超微结构改变。

##### 1.2.5.1 细胞样品制备

取对数生长期的细胞，用0.25%胰酶消化，调整细胞密度至1×10^5^个/mL接种于培养皿（直径35 mm）中，在37 ℃、5% CO_2_培养箱内培养24 h，弃上清，分别加入不同浓度不同粒径二氧化硅DMEM培养基的配制溶液20 mL。分别在37 ℃、5% CO_2_培养箱内培养24 h后，弃去上清液，PBS洗2次，移入Ep管中，1, 500 rpm、10 min，至管底有细胞块状聚沉物，离心后弃上清。3%戊二醛（1/15 mol/L, pH7.4）和1%四氧化锇（0.24 mol/L PBS, pH7.4）各1 mL，固定1 h；系列乙醇脱水，Epon812树脂包埋。

##### 1.2.5.2 连续超薄切片术及电镜观察

取一个旧样品块作为样品托，将样品托的凸出部分用锉刀锉成一个平面，再将Epon812树脂涂在该面上，将上述修好的包埋块粘到样品托上，放入60 ℃烤箱中聚合24 h。用钻石刀在Reichet S型超薄切片机上切连续超薄切片，每张切片厚度为75 nm-80 nm。切片用醋酸双氧铀染色20 min，柠檬酸铅染色7 min。透射电镜观察。

#### 1.2.6 单细胞凝胶电泳实验（彗星实验）检测BEAS-2B细胞处理后DNA损伤情况

用不同粒径的纳米二氧化硅和微米二氧化硅处理BEAS-2B细胞24 h后，弃上清，每孔细胞用0.1 mL PBS漂洗2次，0.25%胰蛋白酶消化，收集2复孔细胞于0.2 mL的PBS中，制成单细胞悬液。取500 μL的0.6%正常熔点琼脂糖铺于单面全磨砂的载玻片上，作为第一层胶，于4 ℃固化5 min；37 ℃下将单细胞悬液与0.75%的低熔点琼脂糖1:1混匀，取100 μL混合液铺于第一层胶上，盖上盖玻片，于4 ℃固化，5 min后揭去盖玻片；取100 μL 0.75%低熔点琼脂糖铺于第二层胶上，于4 ℃固化5 min；5 min后将胶板浸于4 ℃裂解液里裂解1 h；取出胶板，浸于4 ℃电泳液里解螺旋20 min；电泳仪电泳：0.86 V/cm（25 V, 300 mA）4 ℃、20 min。电泳结束后，胶板用Tris-HCl（pH6.8）中和，每张胶板用100 μL的EB染色，盖上盖玻片，于荧光显微镜下观察。启动Comet A 1.0彗星图象智能分析系统进行图像的捕捉与数据的分析，用40倍的物镜进行计数。每个浓度制备两张胶板，每个胶板计数50个细胞（即100个细胞/浓度剂量），计算拖尾率，测量尾长。以Olive尾矩（olive tail moment, OTM）表示DNA损伤程度。

OTM=慧尾DNA含量×尾长

彗星图像判断标准：颜色较亮呈球形的为彗星头部，代表未断裂的、分子量较大的DNA片段；颜色偏暗呈弥散状的为彗尾，代表断裂的、分子量较小的DNA片段。用彗星尾长和彗尾DNA含量的乘积尾矩作为评价指标。

### 1.3 统计学处理

实验数据均采用SPSS 18.0统计软件包进行统计分析。数据进行分析时每组数据先进行正态性检验，如资料服从正态分布或经转换后服从正态分布，则实验数据以Mean±SD表示，组间比较采用单因素方差分析。*P* < 0.05为差异有统计学意义。

## 2 结果

### 2.1 细胞成活率检测结果

经25 mg/mL、50 mg/mL和75 mg/mL的15 nm、30 nm、60 nm和1 µm二氧化硅颗粒物刺激支气管上皮细胞24 h后，发现支气管上皮细胞的成活率随刺激浓度的增加而下降，当刺激浓度为50 mg/mL时，纳米二氧化硅颗粒物组的支气管上皮细胞成活率开始明显下降，分别下降到76.6%、64.1%和87.6%，而1 µm二氧化硅颗粒物组的细胞成活率下降不明显，为90.2%（图 1）。

**1:**
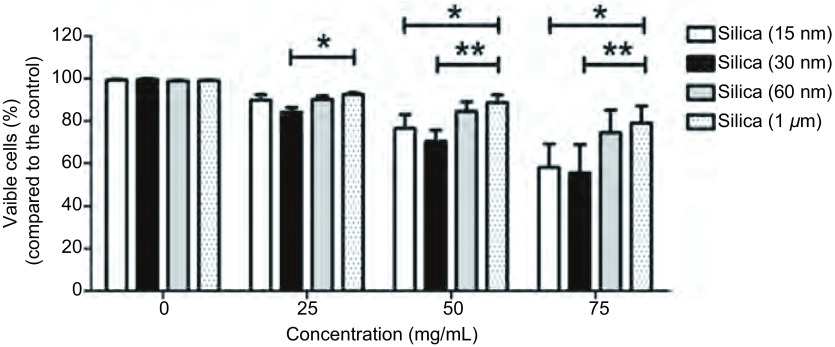
经25 mg/mL、50 mg/mL和75 mg/mL的15 nm、30 nm、60 nm和1 μm二氧化硅颗粒物刺激支气管上皮细胞24 h后的细胞成活率变化，数值采用均数±标准差表示，^*^代表细胞成活率下降组间比较有统计学差异 The cell viability change of BEAS-2B cells after 24 h exposed to 15 nm, 30 nm, 60 nm, and 1 μm silica particles at 25 mg/mL, 50 mg/mL and 75 mg/mL. Values are Mean±SD from three independent experiments, ^*^P < 0.05, ^**^P < 0.01

我们根据细胞成活率的研究结果，进一步研究50 mg/mL的二氧化硅颗粒物刺激支气管上皮细胞24 h时，二氧化硅颗粒物在亚细胞内的分布和对DNA的损伤情况。

### 2.2 透射电子显微镜观察不同粒径二氧化硅细胞内分布

50 mg/mL的二氧化硅颗粒物刺激24 h后，透射电镜观察DMEM组（阴性对照）细胞内外均未发现纳米粒子。细胞表面伸出伪足，形成绒毛状结构。细胞质内有丰富的线粒体、内质网和少数初级溶酶体（图 2A、图 2B）；微米级二氧化硅组细胞质内未发现高电子密度颗粒及吞饮泡，而细胞间质内可见高电子密度颗粒，可见细胞质内线粒体嵴减少，部分线粒体、内质网扩张；核膜完整。部分细胞凋亡，细胞器结构不清，呈块状脱落（图 2C、图 2D）；60 nm二氧化硅组细胞间质和细胞质内可见高电子密度纳米二氧化硅颗粒。细胞质中有多个吞噬泡，内含大量轻度团聚的二氧化硅纳米粒子，位置靠近核膜边缘，有膜样结构，部分膜不完整；可见线粒体嵴减少，线粒体肿胀、滑面内质网、高尔基体扩张；核内异染色质增多。部分细胞凋亡，胞质浓缩，细胞器结构不清，呈块状脱落，可见凋亡小体（图 2E、图 2F）；30 nm二氧化硅组细胞间质可见高电子密度纳米二氧化硅颗粒。细胞质中有多个吞噬泡；内含大量轻度团聚的纳米二氧化硅颗粒，部分位置靠近核膜边缘，有膜样结构，部分膜不完整，边界模糊；包裹在空泡中的纳米二氧化硅颗粒聚积成椭圆形或不规则形团块，密度高于细胞质，与细胞核的电子密度相近。可见线粒体嵴减少，线粒体肿胀、滑面内质网扩张，细胞核肿胀，染色质边集。部分细胞凋亡，细胞形态不规则，胞质浓缩，呈块状脱落，可见凋亡小体。部分细胞核碎裂、溶解，细胞坏死（图 2G、图 2H）；15 nm二氧化硅组细胞间质可见高电子密度团聚纳米二氧化硅颗粒。细胞质内可见多个大吞噬泡包裹着纳米二氧化硅颗粒，边缘模糊，未发现膜样结构。多数靠近细胞核边缘，部分侵蚀细胞核膜。空泡中的纳米二氧化硅颗粒聚积成大片，呈新月形或不规则形等形状，密度高于细胞质，染色深。可见线粒体嵴减少，线粒体肿胀、滑面内质网扩张（图 2I、图 2J）。

**2:**
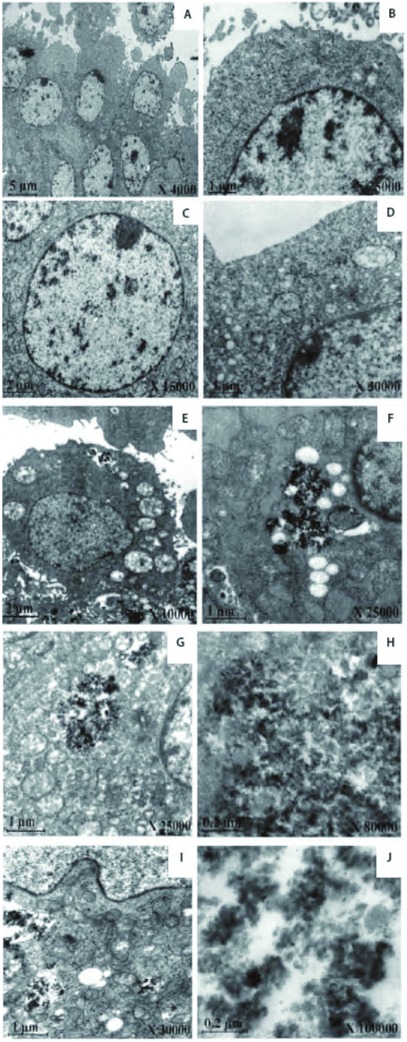
50 mg/mL的二氧化硅颗粒物刺激支气管上皮细胞24 h后的透射电镜图。A、B为对照组，未发现有二氧化硅颗粒物，细胞内有丰富的线粒体；C、D为1 μm二氧化硅颗粒物组，细胞质内未见二氧化硅颗粒物，可见细胞线粒体脊减少；E、F为60 nm二氧化硅颗粒物组，细胞质与细胞间质内可见二氧化硅颗粒物；G、H为30 nm二氧化硅颗粒物组，细胞质内可见二氧化硅颗粒物，部分细胞核碎裂；I、J为15 nm二氧化硅颗粒物组，吞噬泡内可见二氧化硅颗粒物，部分细胞核碎裂 The BEAS-2B cells exposed to Silica particles at 50 mg/mL after 24 h under TEM. A, B are control group, there is no silica particles but abundant mitochondria in cells; C, D are 1 μm silica particles group, there is silica particles in cytoplasm but cristae of mitochondrion reduced; E, F are 60 nm silica group, there is some silica particles in cytoplasm, and some silica particles in interstitial cell; G, H are 30 nm silica particles group, there are some silica particles in cytoplasm, and appear nuclear fragmentation; I, J are 15 nm silica group, there are some silica particles in devour bubble, and appear nuclear fragmentation

经能谱分析发现，细胞吞噬泡内的颗粒和细胞间隙的颗粒均出现一个明显的“硅”峰，提示二氧化硅颗粒物直接或间接地与支气管上皮细胞间发生了作用（图 3）

**3:**
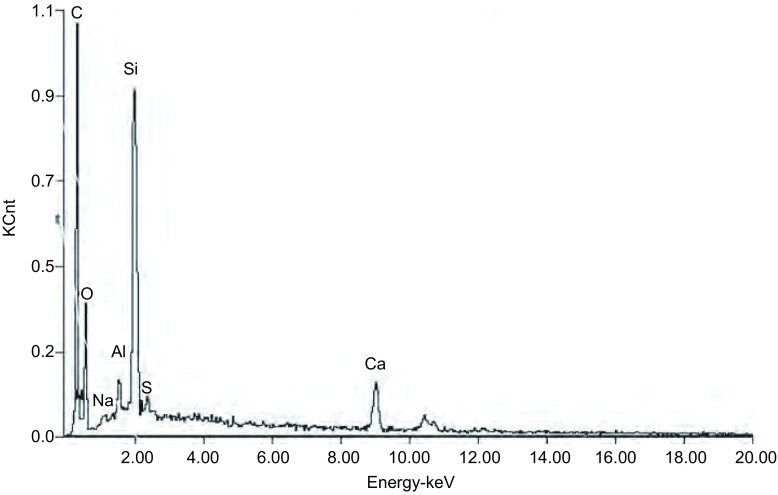
单晶沉积区域EDS分析结果 The EDS analysis of single crystal deposition region

### 2.3 DNA损伤检测结果

荧光显微镜观察可见阴性对照组细胞DNA荧光基本呈圆形，说明DNA损伤较轻；纳米二氧化硅组和微米二氧化硅组DNA均可见荧光拖尾现象，说明均存在DNA损伤情况（图 4）。

**4:**
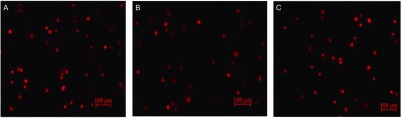
50 mg/mL的二氧化硅处理24 h后，荧光显微镜观察DNA损伤的单细胞凝胶电泳图片。A：DNA荧光呈圆形；B、C：DNA荧光出现拖尾现象 The DNA damage of BEAS-2B cells detected by Single Cell Gel Electrophoresis under fluorescence microscopy. A: DNA fluorescence show circular; B, C: DNA fluorescence show a smearing

### 2.4 对单细胞凝胶电泳图像进行定量分析结果

与对照组比较，BEAS-2B细胞在刺激24 h后，纳米二氧化硅组和微米二氧化硅组的OTM均增加，但以纳米二氧化硅组更明显，差异有统计学意义（*P* < 0.05）；纳米二氧化硅组和微米二氧化硅组比较，OTM均明显增加，差异有统计学意义（*P* < 0.01）（表 1）。

**1:** 不同粒径二氧化硅处理BEAS-2B 24 h后DNA损伤情况（*n*=9） DNA damage of BEAS-2B cells treated by different of silica particles at 24 h (*n*=9)

Group	Counts of cell	OTM (μm/%)
Nano-SiO_2_	105	11.36±1.67^#▲^
Micro-SiO_2_	97	5.93±0.65^#^
DMEM	101	3.28±0.38
BEAS-2B: immortalized human bronchial epithelium cells; OTM: olive tail moment; ^#^: *vs* DMEM group, *P* < 0.05；^▲^: *vs* Micro- SiO_2_ group, *P* < 0.01.		

## 3 讨论

### 3.1 不同粒径二氧化硅在细胞内生物分布机制

纳米颗粒要对细胞产生毒性，必须直接或间接接触细胞。由于纳米颗粒粒径极小，研究^[5-7]^显示，纳米颗粒可通过生物膜上的孔隙或细胞的内吞作用进入细胞及细胞器内，与细胞内生物大分子发生相互作用，破坏生物膜和生物大分子的正常空间结构等。

纳米颗粒到达肺泡后，还可以穿过肺泡上皮细胞而进入肺间质进入血液循环，或者通过淋巴循环进入血液^[8]^，颗粒粒径越小，越容易发生肺外转移，从而越容易进入血液循环，同时，颗粒粒径越小，在血循环滞留时间越长，从而更容易随血液分布到全身各处，对机体可能产生更强的损伤作用^[9]^。因此，有学者提出越小的纳米颗粒越有可能穿透细胞并产生毒性作用^[10]^，但也有学者指出这种说法是片面的，并不是所有的纳米颗粒都能进入细胞，决定纳米物质的生物效应的因素非常多，绝不仅仅局限于尺寸。例如纳米颗粒的剂量、结构、尺寸、表面电荷、表面修饰、聚集状态、晶型等。

### 3.2 不同粒径二氧化硅导致DNA损伤遗传毒性机制探讨

体外细胞实验^[11]^发现，石英能够与DNA发生直接反应。将石英与DNA共同孵育后，石英表面的硅烷醇基与DNA的核苷酸骨架间形成大量氢键。电镜观察到石英粉尘在上皮细胞核内和有丝分裂的纺锤体中存在。推测石英与DNA的直接反应可能对石英的致癌性起决定作用，石英粉尘上的自由基可能干扰了DNA的复制和修复。Yu等^[12]^用无细胞系的实验表明，二氧化硅粉尘可引起λHind Ⅲ DNA链断裂及碱基的氧化。动物实验及细胞培养证明，石英粉尘可诱导氧自由基的产生和DNA链的断裂。物理学方法观察到石英粉尘表面的氧自由基可以与DNA链紧密结合, 并在距石英粉尘攻击的靶碱基数埃的位置对DNA造成损伤^[13]^。傅立叶变换红外光谱观察到：将石英与DNA共同孵育后，石英颗粒表面的硅烷醇基与DNA的核苷酸骨架间形成大量氢键，矽尘可通过氢键而使DNA与粒子表面的二氧化硅结合，形成DNA-矽尘附加物，它可定位于DNA上接近氧自由基产生的部位，进而干预复制、修复和（或）DNA表达以及改变有丝分裂过程，产生突变效应而致癌。

我们对BEAS-2B研究发现，微米二氧化硅不能进入细胞，纳米二氧化硅可以通过细胞膜进入细胞，赋存在细胞质中，部分可见包裹纳米粒子的吞噬泡，部分未见明显的膜样结构，边界模糊。部分包裹纳米粒子的吞噬泡侵犯核膜，但未进入细胞核。与上述研究结果不一致，考虑纳米二氧化硅对不同种类细胞作用可能不同。

细胞膜是细胞与细胞外环境之间的一种选择性通透屏障，既能保障细胞对基本营养物质的吸收摄取、代谢废物的排除和细胞内离子浓度的调节，又能维持细胞相对稳定的内环境。外界物质通过细胞膜转运主要有：胞吞与胞吐、被动运输和主动运输三种方式。纳米颗粒如何进入细胞膜，目前研究尚无定论，多数认为纳米颗粒主要通过细胞吞噬进入细胞。

细胞的被动运输分为简单扩散和协助扩散。简单扩散是胞外的各种极性分子和无机离子，由高浓度向低浓度自由扩散通过生物膜，不需要细胞提供能量。如O_2_、CO_2_、N_2_、水、尿素、葡萄糖、氨基酸、核苷酸及细胞代谢产物等）。其通透性取决于分子的大小、脂溶性和极性。纳米二氧化硅和微米二氧化硅均带负电，不能通过自由扩散通过细胞膜。另外，纳米二氧化硅颗粒虽小，但仍远大于水分子等可自由扩散的分子直径，理论上也是无法自由扩散通过生物膜的。

协助扩散需要特异的膜蛋白载体蛋白和通道蛋白协助完成。主动运输是细胞的选择性运输。纳米二氧化硅和微米二氧化硅不太可能通过以上两种方式进入细胞。

真核细胞主要通过胞吞作用，将大分子与颗粒性物质通过细胞膜移入细胞内。胞吞又分为胞饮作用和吞噬作用，胞饮作用只能转运溶液和小分子物质，形成的囊泡较小（直径一般小于150 nm），大的颗粒物只能通过吞噬作用运输，但吞噬泡的形成需要微丝及其结合蛋白的帮助。由于纳米颗粒的表面效应，进入生物系统的无机纳米粒子会强烈吸附蛋白质分子，甚至改变蛋白质分子的构象^[14]^因此，不排除被纳米二氧化硅吸附的蛋白质分子，向细胞传递生物信号，引起细胞吞噬而进入细胞内。微米二氧化硅由于粒径大，只能由吞噬细胞运输，上皮细胞不能吞噬。

纳米二氧化硅不能进入细胞核，细胞膜具有吞噬作用，但核膜不具此作用，核膜阻挡纳米二氧化硅进入细胞核，但吞噬泡包裹的纳米粒子有向细胞核靠近趋势，可能和纳米二氧化硅带负电有关。

有研究^[15]^发现，纳米二氧化硅可以被摄入细胞，一定条件下能进入细胞核，导致Topo I的异常。Park等^[16]^研究显示，纳米二氧化硅可被吸收至多种细胞的细胞核中，并引起拓扑异构酶I的异常聚集，而微米级二氧化硅只能到达细胞间质，而不能进入细胞质内。这也许是纳米颗粒比微米颗粒造成较大DNA损伤的原因。

本实验发现，纳米二氧化硅比微米二氧化硅造成更大的DNA损伤，与上述研究一致，分析纳米二氧化硅由于其特殊表面效应和小尺寸效应，进入细胞质并靠近细胞核，通过产生大量自由基，直接或间接作用于DNA，导致DNA损伤。

本研究初步得出而个结论：①微米二氧化硅不能进入细胞，纳米二氧化硅赋存在细胞质，二氧化硅的粒径决定其在细胞内的生物分布；②纳米二氧化硅对细胞遗传毒性比微米二氧化硅更严重。
